# Phycocyanin-Loaded Alginate-Based Hydrogel Synthesis and Characterization

**DOI:** 10.3390/md22100434

**Published:** 2024-09-25

**Authors:** Diana-Ioana Buliga, Alexandra Mocanu, Edina Rusen, Aurel Diacon, Gabriela Toader, Oana Brincoveanu, Ioan Călinescu, Aurelian Cristian Boscornea

**Affiliations:** 1 Faculty of Chemical Engineering and Biotechnologies, National University of Science and Technology POLITEHNICA Bucharest, 1-7 Gheorghe Polizu St., 1st District, 011061 Bucharest, Romania; diana_ioana.buliga@upb.ro (D.-I.B.); alexandra.mocanu@imt.ro (A.M.); edina.rusen@upb.ro (E.R.); ioan.calinescu@upb.ro (I.C.); 2 National Institute for Research and Development in Microtechnologies-IMT Bucharest, 126A Erou Iancu Nicolae, 077190 Voluntari, Romania; oana.brincoveanu@imt.ro; 3 Military Technical Academy “Ferdinand I”, 39-49 G. Cosbuc Blvd., 050141 Bucharest, Romania; aurel.diacon@mta.ro (A.D.); gabriela.toader@mta.ro (G.T.); 4 Research Institute, University of Bucharest, ICUB Bucharest, 90 Panduri Rd., 5th District, 050663 Bucharest, Romania

**Keywords:** *Spirulina platensis*, phycocyanin extract, ultrasonic extraction, microwave extraction, release mechanism

## Abstract

Phycocyanin was extracted from *Spirulina platensis* using conventional extraction (CE), direct ultrasonic-assisted extraction (direct UAE), indirect ultrasonic-assisted extraction (indirect UAE), and microwave-assisted extraction (MAE) methods at different temperatures, extraction intervals, stirring rate, and power intensities while maintaining the same algae to solvent ratio (1:15 *w/v*). The optimization of the extraction parameters indicated that the direct UAE yielded the highest phycocyanin concentration (29.31 ± 0.33 mg/mL) and antioxidant activity (23.6 ± 0.56 mg TE/g algae), while MAE achieved the highest purity (Rp = 0.5 ± 0.002). Based on the R_P_ value, phycocyanin extract obtained by MAE (1:15 *w/v* algae to solvent ratio, 40 min, 40 °C, and 900 rpm) was selected as active compound in an alginate-based hydrogel formulation designed as potential wound dressings. Phycocyanin extracts and loaded hydrogels were characterized by FT-IR analysis. SEM analysis confirmed a porous structure for both blank and phycocyanin loaded hydrogels, while the mechanical properties remained approximately unchanged in the presence of phycocyanin. Phycocyanin release kinetics was investigated at two pH values using Zero-order, First-order, Higuchi, and Korsmeyer-Peppas kinetics models. The Higuchi model best fitted the experimental results. The R^2^ value at higher pH was nearly 1, indicating a superior fit compared with lower pH values.

## 1. Introduction

*Spirulina (Arthrospira) platensis* is a cyanobacteria which has been isolated from alkaline saline waters found in tropical and subtropical regions [1,2]. In both natural environments and controlled settings, *Arthrospira* forms spiral-shaped structures called trichomes that vary in size and coil tightness, ranging from tightly wound to straight and uncoiled. These trichomes exhibit distinct transverse cross-walls when observed under the light microscope. Cells within the trichomes are wider than they are long, with widths typically ranging from 3 to 12 µm, occasionally reaching 16 µm. The cellular organization resembles that of a typical prokaryote, lacking membrane-bound organelles [1]. The blue-green alga is a rich source of vitamins (B12 especially), minerals, proteins (comparable amount to the one present in meat or soybeans), and carotenoids [3,4,5]. Its specific colouration is derived from the presence of chlorophyll and phycocyanin.

Phycocyanin is a light-harvesting pigment-protein complex. It has a blue hue and has been shown to have antioxidant [5,6,7], anticancer [8,9], and anti-inflammatory properties [10]. It can also be employed as a natural blue functional food colourant [10] or as cosmetic dye [7]. Phycocyanin is a hydrophilic protein containing a fluorescent pigment that can be categorized into three groups: phycocyanin (dark blue), phycoerythrin (red), and allophycocyanin (light blue). Its primary function is to transfer excitation energy to the reaction centre, where the maximum absorption wavelength is around 620 nm [4]. 

Several extraction methods have been reported and all are influenced by temperature, pH, solvent type, vegetal material/solvent ratio, and if either fresh or dried biomass was used [11]. The influence of temperature is due to cell membrane modifications which lead to an increase mass transfer rate. With increasing temperature, there is a higher extracted amount of interest compound. However, over 50 °C, a decrease in the extracted concentration arises as denaturation of the protein structure occurs [12,13]. This results in modifications of the chromophore stability and, subsequently, colour change. According to Ferreira-Santos et al. [14], the degradation may occur due to modifications on the quaternary, tertiary, and secondary structures. Moreover, a high temperature aids the extraction of cytoplasmatic proteins and other interfering compounds, reducing the purity of phycocyanin extract [15]. The pH is another important aspect to consider. The isoelectric point of phycocyanin varies depending on species and environmental factors during the growing stage but generally has values between 4.1 and 6.4 [16]. Near these pH values, the extraction is more difficult because the protein interacts less with water. At pH values lower than 5, the purity is also decreased [17]. As extractive solvents, the use of sodium-phosphate buffer, distilled water, NaCl solution, and CaCl_2_ solution were reported as similar [11]. According to Jaeschke et al. [11], frequently used vegetal material to solvent ratios are 1:25, 1:20, 1:15, and 2:25. Regarding the type of raw material, it is still an uncertainty if there is a significant impact on the results when fresh or dried alga is utilized. Nonetheless, it has been shown that a prior hydration of the dry vegetal material leads to better extraction yields compared to a direct use of the dry powder [18]. 

As extraction methods, several are reported in literature. Cycles of freezing and thawing are used to disrupt cell membranes. During freezing, the expansion of intracellular water causes the cell volume to increase, altering pressure conditions and damaging the membrane. Subsequently, during thawing, the cell contracts [11,19,20,21], mixing and homogenization [15,22,23], bead milling [24,25], high pressure processing [26], pulsed electric field [25,27], microwaves [23,28], and ultrasounds [18,29,30,31] being extraction methods applied for obtaining phycocyanin. 

Its antimicrobial property [32] could be exploited into a drug delivery system (DDS). DDS are known to provide prolonged and controlled drug release over an extended period [33,34], being a potential solution for accelerating wound healing. The active substance is administered via chemical diffusion or penetration at a stable rate and a suitable concentration [35]. The therapeutic efficiency of the compound is influenced by the controlled-release drug carrier. Depending on the type of carrier or the system conditions, the delivery characteristics can vary [36]. As a result, it is necessary to develop special slow-release drug carriers. 

Polymer-based hydrogels are a feasible and practical option for drug delivery systems. Their properties—porosity [37,38,39], high water content [40,41], soft texture—make them suitable for biomedical applications [33,42,43]. Depending on the composition, hydrogels are synthetic (based on polyacrylic acid (PAA), polyacrylamide (PAM), polyvinyl alcohol PVA, etc.) or of natural origin (collagen, chitosan, hyaluronic acid, alginate) [35]. The biobased hydrogels have the advantage of good biocompatibility and biodegradability, while the synthetic ones are more precise, easier to control and modify [35]. Depending on applications and type of crosslinking (chemical or physical) a combination of both natural and synthetic starting materials can be utilized. 

In drug delivery and wound dressing applications PVA is a synthetic semi-crystalline polymer that meets critical requirements such as biocompatibility, biodegradability, ease of processing (being water-soluble), non-toxicity, increased oxygen permeability, high water retention capacity, bio-adhesiveness, and low cost [44,45]. PVA offers a new perspective on pharmaceutical products and tissue engineering regeneration by enhancing drug solubility, absorbing large amounts of water while maintaining necessary moisture levels, and increasing oxygen permeability, which accelerates wound healing and facilitates drug release [46]. However, controlled drug delivery is often achieved by combining PVA with various natural polymers such as alginate, chitosan, pullulan, or bacterial cellulose [47,48,49,50].

Alginate is the most abundant natural biopolymer after cellulose being extracted from different species of brown seaweeds mostly used in wound dressing and tissue engineering due to biocompatibility, biodegradability, low toxicity, low cost of extraction process, and good 3D foam, sponge or hydrogel-forming structures [51,52]. 

Therefore, the primary purpose of this study was to identify the optimal extraction method for phycocyanin from *Spirulina platensis*, focusing on maximizing concentration, antioxidant capacity, and purity by applying three different extraction techniques (conventional, microwave-assisted and ultrasonic-assisted processes). The secondary purpose was to develop polymer-alginate-based hydrogels for the controlled release of the phycocyanin extract. 

The hydrogels from this study were developed by employing thermal radical polymerization of acrylamide (AM) in the presence of PVA and alginate cross-linked by N, N’-methylene-bis-acrylamide (MBSA) triggered by potassium persulfate as initiator. 

The hydrogels were lyophilized and characterized by FT-IR, SEM, and compression tests. Considering the importance of kinetics in drug delivery systems, the phycocyanin’s release profiles were obtained at two different pH values (7.45, respectively 6.5) and discussed based on Zero-order, First-order, Higuchi model and Korsmeyer-Peppas model which are the most suited to describe the drug release mechanism from polymeric matrix [53,54].

## 2. Results and Discussion

### 2.1. Extraction of Phycocyanin

For the first part of the study, phycocyanin was extracted from *Spirulina platensis* in both conventional and unconventional processes.

Phycocyanin content was determined using Equation (1), obtained as described in Section 3.3.1.

(1) $${Phycocyanin}_{conc}=\frac{{Abs}_{618}}{1.8665},$$

where: Phycocyanin_conc_ is the concentration of phycocyanin expressed as mg/mL and Abs_618_ is the absorption value corresponding to 618 nm.

**Figure 1:**
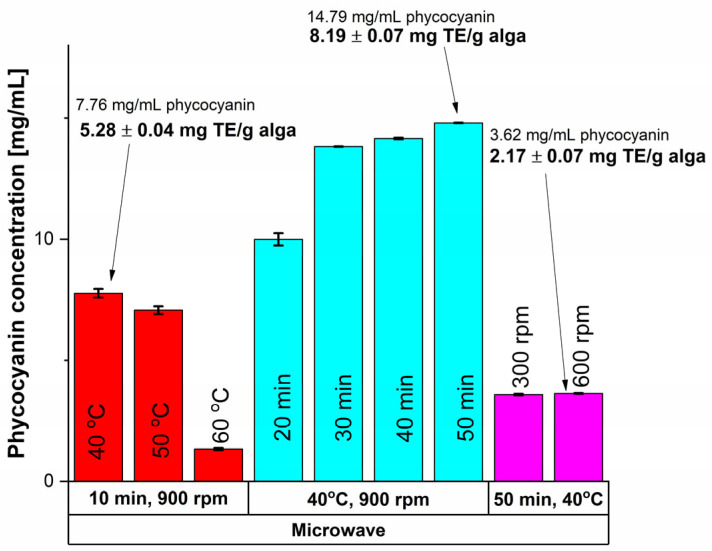
Influence of time, temperature and rotation speed on the extracted phycocyanin content using MAE and on the antioxidant activity.

MAE was one of the unconventional extraction methods utilized and the results are shown in Figure 1.

Based on the results shown in Figure 1, higher values of antioxidant activity, assessed after the CUPRAC (CUPric Reducing Antioxidant Capacity) assay as mg Trolox equivalents (TE)/g alga—methodology presented in Section 3.3.2, were obtained at lower temperatures, namely 40 °C compared with 60 °C, which is explained by the thermo-sensitivity of the phycocyanin compound [55]. The influence of time was also studied, and a 90.61% increase in the phycocyanin amount was recorded when a 50 min treatment was applied, compared to only 10 min at 40 °C. To ensure a good homogeneity of the mixture, efficient stirring is important, as it provides a good contact between the solvent and the algae material. Consequently, the stirring rate influenced the antioxidant activity of the interest compound and for a MAE at 40 °C and 50 min, there was not a significant difference between the stirring rate of 300 rpm compared with 600 rpm. Thus, the highest values in terms of phycocyanin content and antioxidant activity were registered at a stirring rate of 900 rpm.

To conclude, considering MAE, for a 14.79 ± 0.07 mg/mL concentration an antioxidant activity of 8.19 ± 0.07 mg TE/g algae was obtained, which was achieved at a temperature of 40 °C, a stirring rate of 900 rpm, and an extraction time of 50 min.

Nevertheless, another important aspect to consider is the purity ratio (R_P_) calculated as described in Equation (2), according to Liu et al. [56].

(2) $$R_{P}=\frac{{Abs}_{620}}{{Abs}_{280}},$$

where: R_P_ is the purity ratio of phycocyanin, Abs_620_ is the absorption value corresponding to 620 nm, Abs_280_ is the absorption value corresponding to 280 nm.

For a 30 min treatment, the purity ratio (R_P_) had a value of 0.5 ± 0.002, significantly higher compared with 0.39 ± 0.000, and 0.40 ± 0.002 resulted for 40, respectively 50 min. Thus, the best results in terms of phycocyanin concentration, antioxidant activity and purity of the extract detailed also in Table 1 (13.82 ± 0.01 mg phycocyanin/mL extract, 5.67 ± 0.11 mg TE/g algae, R_P_ = 0.5 ± 0.002) were obtained for a MAE of 30 min, 40 °C and 900 rpm. 

**Figure 2:**
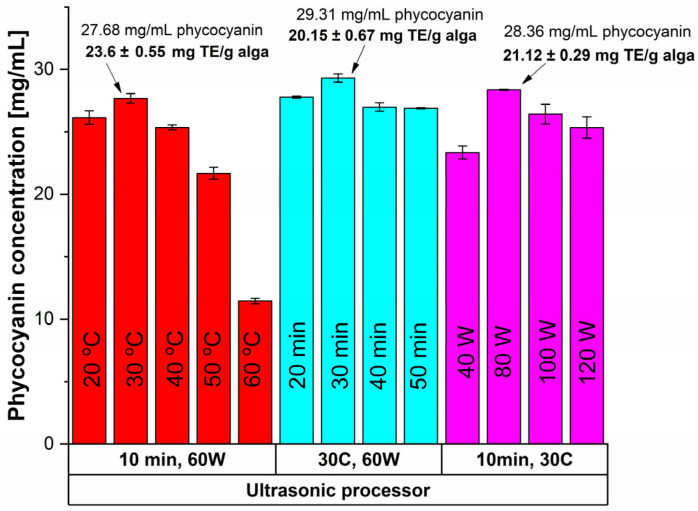
Influence of time, temperature and power input on the extracted phycocyanin content and on the antioxidant activity (expressed as mg TE/g alga) using an ultrasonic probe in continuous mode.

**Figure 3:**
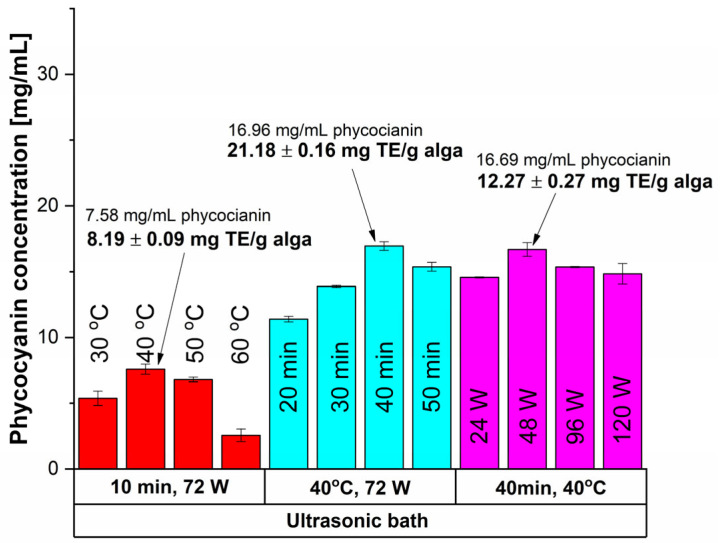
Influence of time, temperature and power input on the extracted phycocyanin content and on the antioxidant activity (expressed as mg TE/g alga) using an ultrasonic bath.

The UAE was performed using an ultrasonic probe paired with a temperature control unit (direct UAE) and an ultrasonic bath (indirect UAE); the results are shown in Figure 2 and Figure 3, respectively.

In the first case (shown in Figure 2), the effects of time, temperature, and power input on continuous sonication were investigated. For a 10-min treatment, the highest extraction yield (27.68 mg/mL) was achieved at 30 °C with an R_P_ of 0.37 ± 0.007 (Table 1). When evaluating the impact of these parameters, the best results were observed after 30 min of sonication (Figure 2).

To optimize energy efficiency, power variation was tested for 10 min at a constant temperature of 30 °C. While increasing power from 60 W to 80 W slightly improved the phycocyanin concentration by 2.45%, the 30% rise in energy consumption did not justify this small gain. As a result, the optimal extraction conditions were determined to be a 30-min ultrasound treatment at 30 °C and 60 W, yielding an R_P_ of 0.45 ± 0.003 and a phycocyanin concentration of 29.31 ± 0.33 mg/mL.

Using the same equipment, the UAE was performed in discontinuous mode considering a sonication cycle of 1 s ON and 1 s OFF, for 10 min, at 30 °C, and 60 W. The results were similar to the previous case: the phycocyanin concentration decreased by 23.40%, and the R_P_ value decreased by 17.78%. Meanwhile, the antioxidant activity value remained unchanged (see Table 2). Therefore, a good compromise could be assessed regarding the concentration and purity of phycocyanin in favor of high antioxidant activity and significantly lower power input. 

The UAE performed in a sonication bath (indirect UAE) exhibited poorer outcomes (Figure 3) than the previous case, pointing to the milder nature of this extraction method.

For a 10 min sonication time at 72 W, the highest results for phycocyanin content (7.58 mg/mL) and antioxidant activity (8.19 mg TE/g alga) were registered at 40 °C. Higher temperatures led to reduced concentrations due to thermal degradation. By increasing the time extraction to 40 min, a higher concentration of phycocyanin and antioxidant activity were obtained. In terms of applying lower sonication power (48 W) a slight decrease in phycocyanin concentration was registered (1.55%) but with a significant drop of 42.08% of antioxidant activity. Thus, the optimal conditions for indirect UAE were registered at 40 min, 40 °C, and 72 W as mentioned in Table 1.

**Figure 4:**
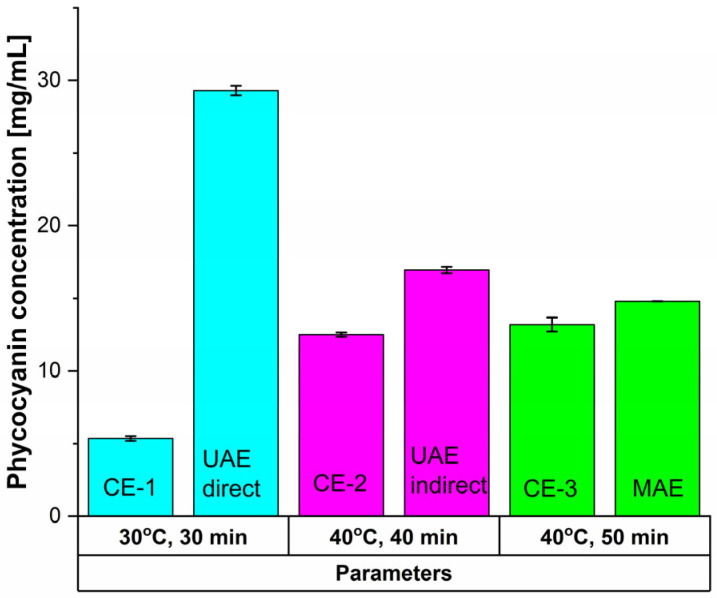
Influence of time and temperature on the extracted phycocyanin content using CE methods and comparison with the unconventional equivalents.

Compared to the previously presented cases, the conventional extraction methods (Figure 4, Table 2) led to even poorer results.

For CE-1, after 30 min at 30 °C and 900 rpm, phycocyanin concentration registered a 81.71% decrease compared with UAE direct. Antioxidant activity dropped by 85.06%, and R_P_ by 46.67%. For CE-2 (40 min at 40 °C and 900 rpm), concentrations and antioxidant activity were 26.31%, respectively 77.42% lower than indirect UAE, but R_P_ increased by 18.75% (0.38 ± 0.002). Compared to MAE (50 min at 40 °C), CE-3 showed a 10.81% reduction in phycocyanin concentration, a 35.82% loss in antioxidant activity, and a 4.28% decline in Rp.

To conclude, the evaluation of the obtained results (see Table 1) reveals that, for the used extraction methods, the highest phycocyanin amount was recorded for the direct UAE employed in the continuous mode. Overall, regarding the amount of extracted interest compound, after the ultrasonic probe (continuous and 1 s ON 1 s OFF) are the indirect UAE, MAE, and CE respectively.

For direct sonication, the 1 s ON 1 s OFF treatment yielded lower amounts than continuous mode but showed better efficiency in terms of treatment time and energy. Compared to UAE and other studies, we have obtained lower concentrations, but the purity of the extract was higher compared to results reported by Gorgich et al. (0.34 ± 0.01) [57]. Other studies reported higher concentrations (e.g., 74.51 mg/g and 115 mg/g) but considerably lower purities of 0.56 [21,58]. Indirect sonication had lower yields than direct extraction due to ultrasound energy loss through the sonication medium and reactor walls, as noted by Sukor et al. [59] and Aznar-Ramos et al. [60]. 

Close concentrations result for MAE and the indirect UAE. MAE provides increased selectivity in extractions as compared to UAE, where sonication promotes asymmetric bubble collapse. 

This is the key factor in UAE and it generated jets of solvent towards the vegetal material which improves the extraction efficiency [61]. Due to this element, the UAE could be more powerful than the MAE, meaning that other antioxidant-rich compounds could have been extracted, such as chlorophyll, throughout the process. 

In our case, the CE methods are not as efficient as the unconventional ones, leading to poorer results. Although in Table 1 the conventional and MAE results are similar, the time increase of 66.67% necessary for CE is not energetically and temporally feasible. Similar behavior is reported by Sukor et al. [59], and Minchev et al. [31]. for the phenolic acids extraction.

On the extracts obtained as described in Table 1 were also performed FT-IR analyses.

**Figure 5:**
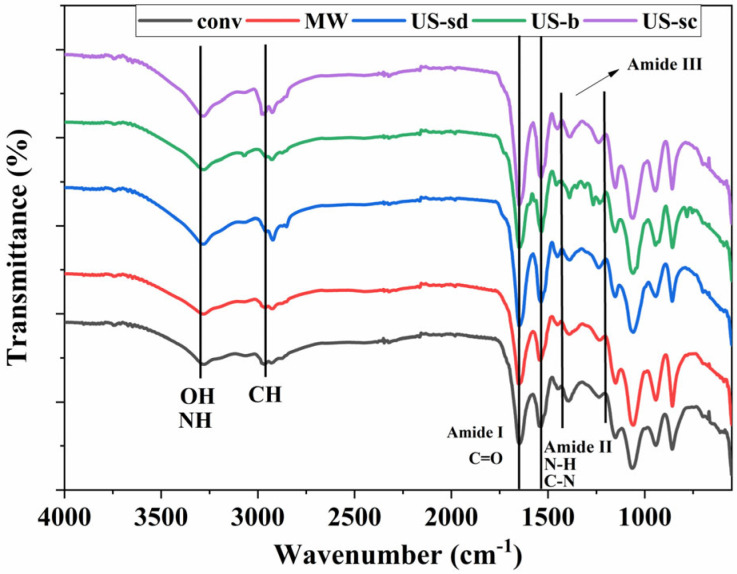
FT-IR spectra of the extracts (conv—conventional, MW—MAE, US-sd—1 s ON 1 s OFF direct sonication, US-b—indirect sonication, US-sc—continuous direct sonication).

The FT-IR spectra of the extracts presented in Figure 5 display the presence of the three typical protein bands of amide I (C=O stretching vibrations) (1653 cm^−1^), amide II (N-H and C-N deformation) (1538 cm^−1^), and amide III (in the range 1200–1400 cm^−1^) all corresponding to literature data in which phycocyanin was extracted from *Spirulina platensis* specie [62,63,64,65]. The maximum transmittance peak specific for phycocyanin is recorded at 1645 cm^−1^ matching with amide I vibrations being in good accordance with literature data Al-Malki [66]. Also at 2960 cm^−1^ CH stretching from CH_2_, and CH_3_ groups was recorded [65]. The CH_2_ bending vibration was also visible at 1424 cm^−1^ in all cases, while the absorption peaks at 3400–3500 cm^−1^ specific for N-H and O-H stretching [65,66] are more clearly developed for the extracts obtained under ultrasound-assisted conditions.

### 2.2. FT-IR Analysis of the MW Extract and of the Polymer-Alginate-Based Hydrogels

**Figure 6:**
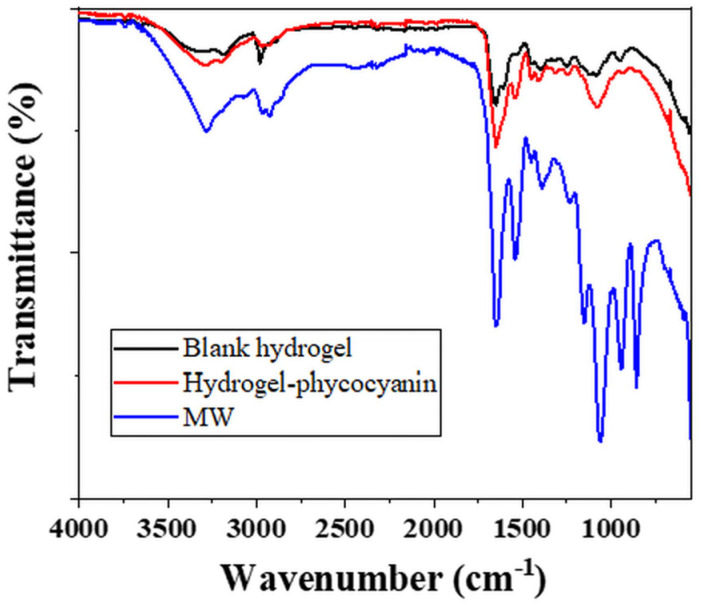
FT-IR analysis of the blank, phycocyanin loaded hydrogels and MW extracted phycocyanin.

The hydrogels obtained as described in Section 2.3 and prepared as presented in Section 2.4 were analysed by FT-IR (Figure 6) before and after the absorption of phycocyanin extract obtained through MAE according to the conditions outlined in Table 1.

The blank hydrogel (Figure 6—black line) recorded a first peak at 3313 cm^−1^ which can be attributed to the -OH stretching from PVA or NH stretching from the PMBSA, the cross-linking agent [67,68,69]. The next signal indicates -CH stretching vibrations at 3184 cm^−1^, while at 2979 cm^−1^ asymmetric stretching of CH group was registered [67,68,69]. The characteristic peak for carbonyl group C=O (from amide I band) specific to PAM was evidenced at 1652 cm^−1^, while the NH bending from PAM and cross-linker appeared at 1602 cm^−1^ [69,70]. Nevertheless, at 1602 cm^−1^, the O-C-O carboxylate asymmetric groups of alginate compound can be registered [70]. The signal from 1535 cm^−1^ was assigned to -NH bending vibrations coupled with C-N stretching (specific for amide II band) in cross-linker chains [68,69]. At 1402 cm^−1^ the CN stretching group can be attributed to PAM on one hand and to the C-OH deformation vibration with the contribution of the symmetric stretching vibration of O-C-O in alginate compound [68,69,70]. The crystalline sequence of PVA was evidenced by the registration of a signal at 1119 cm^−1^ corresponding to the stretching of C-O group [67]. At 1080 cm^−1^ the C-C asymmetric stretching from PAM or PMBSA [68,69].

For the hydrogel loaded with phycocyanin (Figure 6—red line) the presence of the extract was quite clear by the modification in intensity and position of the signals from 3290–3190 cm^−1^ (specific for OH and NH stretching), 1651 cm^−1^ (corresponding to amide I), 1535 cm^−1^ (attributed to amide II) confirming the modification of the polymer-alginate based compounds.

### 2.3. Scanning Electron Microscopy of the Blank Hydrogels and Loaded Hydrogels with Phycocyanin

**Figure 7:**
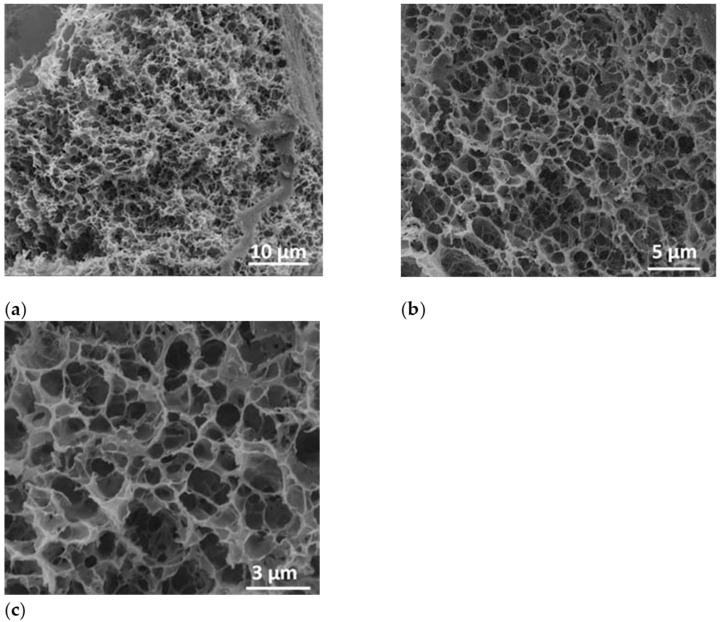
SEM images of polymer-alginate-based structure at a magnification of 5000× (**a**), 10000× (**b**), and 20000× (**c**).

After the lyophilization process, the sample was fractured and investigated by SEM at a tilt angle of 0° to prove the formation of porous structure inside the polymer-based specimens. Thus, in Figure 7, the micrograph images indicated a highly porous characteristic of the polymer-alginate-based structure after water removal by the lyophilization process. In Figure 7a, as well as in the detailed micrographs (Figure 7b,c) a highly porous structure of the lyophilized sample was registered.

The size distribution of the polymer-alginate lyophilized hydrogel was obtained from SEM images by measuring around 300 nanopores. The nanopore sizes were found to be ranged from 153 nm to 1.53 µm. The histogram was best fitted with Gauss function. The highest percentage of the hydrogel pores were found to be within 423 nm–1.06 µm diameter size range having the mean diameter 707 ± 105 nm (Figure S2).

### 2.4. Compression Tests for the Polymer-Alginate-Based Hydrogels

**Figure 8:**
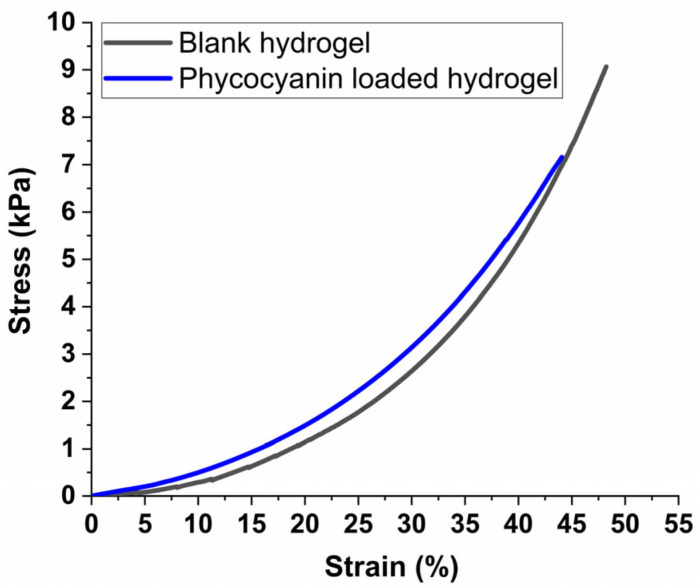
Stress-strain plots for equilibrium swelled hydrogels submitted to compression tests.

Figure 8 presents the stress-strain plots of equilibrium-swelled hydrogels under uniaxial compression tests. These plots show the relationship between stress and strain, which helps determine the mechanical properties of the hydrogels under compression [71]. This information is useful in designing materials for various applications, such as tissue engineering and drug delivery systems [72]. It can be observed from the comparative graph (Figure 8) that the mechanical properties of the hydrogel suffer only minor modification due to the introduction of the phycocyanin, both materials enduring strains up to around 50% before reaching their stress limit. This implies that the maximum strain of the phycocyanin-loaded hydrogel was only slightly reduced, which is consistent with literature research on PAM-alginate-based hydrogels that registered reduced mechanical performance upon swelling compared with unloaded samples [73].

It is worth mentioning that at the maximum strain of 50%, the maximum stress in our case reached 9040 Pa for the blank sample, respectively 7136 Pa for the loaded hydrogel which is almost 3 times higher compared with literature data in which polyacrylamide-alginate-based hydrogels were designed to mimic muscle tissue [73]. Also, as demonstrated by Darnell et al. [74], by increasing the concentration of the cross-linker in polyacrylamide-alginate-based hydrogels, a compressive strain of up to 90% can be reached without any structural modifications (cracks or deformations) to the materials, making them suitable for vascular improvement applications.

**Scheme 1:**
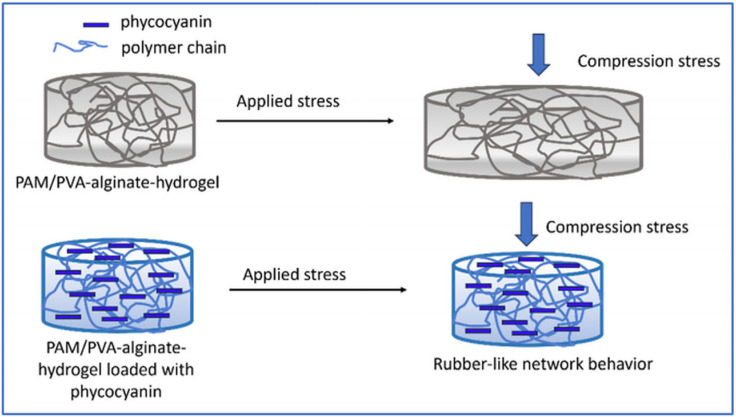
Rubber-like behavior of the polymer-based hydrogel loaded with phycocyanin.

Thus, in our study, the observed compressive results can be attributed to the higher concentration of cross-linker compared with literature date, which likely contributes to a stiffer structure, providing higher rigidity giving the possibility to tailor the formulation of hydrogels in accordance with different applications. On the other hand, the encapsulation/incorporation of pyocyanin into the hydrogel’s pores led to a rubber-like network behavior preventing the deformation of the sample (Scheme 1), further enhancing the stiffness of the structure.

This resulted in a lower compressive strain observed at the same level of compressive stress which can be attributed to the conformational structure of phycocyanin that is the result of a self-assembling process of trimeric aggregates [75,76].

On the other hand, the highly porous nature of the hydrogel demonstrated by SEM analysis and statistics for pore size determination (Figure 7, respectively Figure S2), adds flexibility to the structure. This combination of stiffness from the cross-linker and flexibility from the porous structure can result in particular mechanical properties that may be advantageous for various applications that request structural integrity like drug delivery systems and products that conform to body contours [77,78,79].

### 2.5. Controlled Delivery of Polymer-Alginate-Based Hydrogels

To characterize the release profile of the polymer-alginate-based hydrogels 4 mathematical models were considered to analyse the phycocyanin release such as Zero-order kinetics, First-order kinetics, the Higuchi model, and the Korsmeyer-Peppas model considering that these are the most used kinetic models for the drug release mechanism from polymeric matrix [53,54,80].

**Figure 9:**
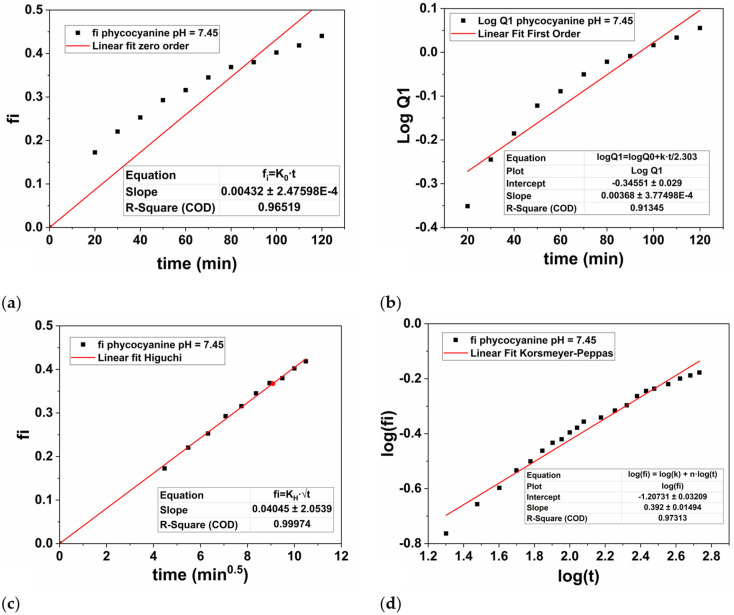
Graphical representation of zero-order kinetics (**a**), first-order kinetics (**b**), Higuchi kinetics (**c**), and Korsmeyer-Peppas kinetics (**d**) for the phycocyanin release at pH = 7.45.

**Figure 10:**
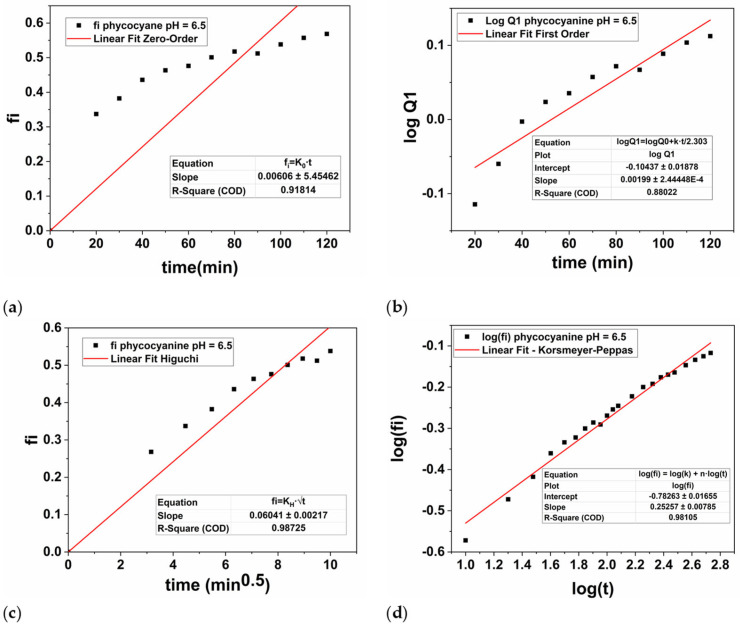
Graphical representation of zero-order kinetics (**a**), first-order kinetics (**b**), Higuchi kinetics (**c**), and Korsmeyer-Peppas kinetics (**d**) for the phycocyanin release at pH = 6.5.

Therefore, the hydrogel discs loaded with phycocyanin were subjected to controlled release for 9 h at two different pH values, namely 7.45, and 6.5. The experimental results were plotted based on Zero-order, First-order, Higuchi’s model and Korsmeyer-Peppas and presented in Figure 9 (pH = 7.45), respectively Figure 10 (pH = 6.5).

To better explain the differences in the values recorded by the parameters of each model, mathematical calculations were performed to determine the angular and linear coefficients for the linearized equations of all kinetic models. To provide a clearer understanding of the release process and to examine the kinetics of phycocyanin release, the parameters for each model were summarized in Table 3.

For the parameters of the First-order equation the angular coefficient is k_1_/2.303 (slope value) and the linear coefficient is logQ_0_ (intercept value). Thus, the values of k1 were obtained by multiplying the value of the angular coefficient with 2.303, while the value of Q_0_ was obtained by applying the inverse of the logarithm, namely 10 to the power of the intercept value −0.3455 (Figure 9b), respectively −0.1043 (Figure 10b). Thus, in Table 3 the values of k1, respectively Q_0_ were presented for both cases.

For the Korsmeyer-Peppas model, the parameters k, and n were obtained from the linearized equation of the power law model (Equation (3)) at pH = 7.45 (Figure 9d), respectively pH = 6.5 (Figure 10d):

(3) $$\log\left(f_{i}\right)=\log\left(k\right)+n\cdot log(t),$$

In this case, from mathematical point of view, the n parameter is the slope of the linearized equation, while log(k) is the intercept. Thus, the values of k were obtained by raising 10 to the value of the intercept −1.2073 displayed in Figure 9d, respectively −0.7826 in Figure 10d.

In our study, the phycocyanin release performed at a pH of 7.45 (Figure 9) was best described by the Higuchi model, as evidenced by the highest regression coefficient (R^2^ = 0.9997) compared to the other models: Zero-order (R^2^ = 0.9651), First-order (R^2^ = 0.9131), and Korsmeyer-Peppas (R^2^ = 0.9731). When the pH value was decreased to 6.5, the Higuchi model again provided the best fit with an R^2^ of 0.9872 (Figure 10c), compared to Zero-order (R^2^ = 0.9181), First-order (R^2^ = 0.8802), and Korsmeyer-Peppas (R^2^ = 0.9811). However, the R^2^ value at the higher pH was significantly higher compared with the pH value of 6.5, being very close to 1, which indicates a superior fit. According to the Higuchi’s model, the polymer-based system is characterized by a hydrated layer that is expected to emerge on the surface’s edge where the release medium is present as based on a Fickian’s diffusion process [81]. Overall, as the hydrogel’s contact time with the medium increases, the active compound that has been encapsulated/loaded into the PAM/PVA-alginate matrix (in our case, phycocyanin) diffuses toward this layer and dissolves into the medium [82].

It is worth mentioning that at lower pH values, the release constant of Higuchi model, K_H_ is higher compared with the value obtained at higher pH, namely 0.0604 at pH = 6.5 compared to 0.0404 at pH = 7.45. This behavior of the PAM/PVA-alginate based system is attributed not only to the highly porous structure of the hydrogel, but also to the phycocyanin solubility in the release medium during the drug delivery experiments [55,83,84,85].

Considering that we used the same polymer matrix and we preserved the same temperature while phycocyanin was released, it is worth mentioning that the solubility of the phycocyanin at different pH values is the key factor that influenced our experiments that is strongly connected with the phycocyanin’s structure. Thus, according to Falkeborg et al. [84] and Debreczeny et al. [83] at pH values higher than 4 the conformation of phycocyanin compound is not altered. Typically, at pH values below 4, phycocyanin adopts a folded conformation as a consequence of the denaturation of its protein subunits, which alters its absorption properties turning it from blue to green [55].

However, at pH values above 6, phycocyanin maintains its unfolded structure, and the polymer-alginate hydrogel is not affected by conformational changes of the absorbed compound. In our case, more amino acid residues coming from the protein subunits become protonated at lower pH values (6.5 compared with 7.45), resulting in an overall increase in the positive charge of the protein part [55,85]. Additionally, pH 6.5 the structure of phycocyanin could be partially unfolded which exposes more the hydrophilic groups to the aqueous release medium [85].

Thus, our results are in good agreement with literature data which confirms that phycocyanin compound does not significantly degrade or alter, its structure being quite stable within a pH range of 5.5 to 8 and up to a temperature of 45 °C [55]. Thus, in this study, the solubility of phycocyanin increases at lower pH values, accelerating the diffusion process consequently leading to a higher concentration of phycocyanin being released.

To summarize, based on our results and comparison with literature data, the drug delivery process is therefore influenced by the solubility of the phycocyanin that is higher at lower pH values of the release medium. These findings suggest that at lower pH levels, the release rate and concentration of phycocyanin are higher, which is generally not ideal for prolonged controlled drug release. In practice, controlled release is more advantageous at higher pH levels in our case, as phycocyanin will be released more slowly, thereby ensuring a more extended-release period of the active compound.

## 3. Materials and Methods

### 3.1. Materials

The used powdered spirulina was purchased from a local market: Spirulina platensis (lot 200616) distributed by Herbal Sana^®^ and Blue Spirulina Powder (lot 23163) marketed by Dragon Superfoods^®^. Phosphate buffer solution (PBS) was utilized as extractive solvent, while neocuproine (98%, Sigma-Aldrich, Taufkirchen, Germany), copper (II) chloride (99%, Sigma-Aldrich), ammonium acetate (97%, Sigma-Aldrich), and 6-hydroxy-2,5,7,8-tetramethylchroman-2-carboxylic acid (TROLOX) (97%, Sigma-Aldrich) were used for the CUPRAC assay. For the alginate-based hydrogels synthesis polyvinyl alcohol (PVA, average Mw = 85,000 − 124,000 g/mol, 87–89% hydrolyzed), sodium alginate, acrylamide (AM), N,N′-methylenebis(acrylamide) (MBSA), and ammonium persulphate (APS), all from Sigma-Aldrich were used as received. APS was purified by recrystallization from ethanol.

### 3.2. Methods

#### 3.2.1. Extraction of Phycocyanin

Conventional and unconventional extraction methods were employed. For all situations, an alga to solvent ratio of 1:15 (*w/v*) was used. All the extractions but the microwave-assisted (MAE) ones were performed in a 50 mL jacketed vial with the same magnetic stirrer and a pre-stirring of 1500 rpm for 1 min. The MAEs were performed in a special 20 mL vial and had a 1-min pre-stirring at the corresponding rpm value set for the whole extraction. The obtained extracts were stored prior to analyses at 4–8 °C after a 15 min centrifugation at 4000 rpm. Two extraction intensification techniques were used, namely microwave and ultrasound (bath and probe) assisted extractions. The MAEs were performed using the Biotage^®^Initiator microwave applicator (Biotage, Uppsala, Sweden). The temperature (40, 50, 60 °C), time (10, 20, 30, 40, 50 min), and stirring rate (300, 600, 900 rpm) were varied. When the ultrasonic bath was utilized, the power (24, 48, 72, 96, 120 W), time (10, 20, 30, 40, 50 min), and temperature (30, 40, 50, 60 °C) were altered. For the Hielscher UP200H ultrasonic titanium probe (7 mm diameter) (Hielscher, Teltow, France), the same parameters were varied: temperature (20, 30, 40, 50, 60 °C), time (10, 20, 30, 40, 50 min), and US power (40, 80, 100, 120, 240 W). In this case, the temperature was maintained aided by the Labo S200-H13 (Labo, Guangzhou, China) bath with internal and external recirculation with cooling and heating function. For example, the aspect of the phycocyanin extract obtained by MAE is presented in Figure S1a. For the conventional extractions (CEs) similar conditions to the ones utilized for MAE and UAE (bath—indirect and probe—direct) were employed. The parameters were selected based on the obtained results, mainly the highest phycocyanin concentration. The stirring rate was the same for all CEs with a value of 900 rpm. The used reactors were the same as the ones utilized for MAE and UAE, respectively.

#### 3.2.2. Synthesis of Polymer-Alginate-Based Hydrogels

10 mL of distilled water, 0.1 g of PVA, and 0.15 g of sodium alginate were added to a 15 mL vial. In the aqueous solution were added 0.5 g of AM, and after complete dissolution, 0.05 g of MBSA was introduced as cross-linking agent. After adding 0.1 g of APS, the reaction was run for two hours at 60 °C.

#### 3.2.3. Loading of Polymer-Alginate-Based Hydrogels with Phycocyanin and Controlled Delivery Conditions for Phycocyanin

The hydrogels previously obtained as described in Section 3.2.2 were cut into discs of 5.44 ± 0.55 mm height and 12.78 ± 0.29 mm diameter and lyophilized following the next steps: (a) sample freezing at −18 °C for 24 h; (b) freeze-drying for the next 24 h in the BIOBASE BK-FD10S equipment (Biobase, Jinan, China) with a condensation capacity of 1 kg/12 h, at −60 °C and 10 Pa. The aspect of the resulted hydrogel and lyophilized hydrogel is depicted in Figure S1b, respectively Figure S1c.

0.075 ± 0.002 g of lyophilized hydrogels were kept in the presence of 15 mL of phycocyanin extracts obtained by MAE (extraction conditions: 1:15 *w*:*v* algae to solvent ratio, 40 min, 40 °C, 900 rpm) for 24 h (Figure S1d).

The maximum swelling ratio (SR) was calculated based on Equation (4) adapted after Saraydin et al. [86]:

(4) $$Swelling\;ratio(\frac{g}{g})(SR)=\frac{w_{1}-w_{0}}{w_{0}},$$

where: w_0_ is the weight of lyophilized gel (expressed in grams), and w_1_ is the maximum weight of swollen lyophilized gel (expressed in grams) reached after 24 h. The SR registered around 10.45 ± 0.49 g/g.

### 3.3. Characterization Methods

#### 3.3.1. Phycocyanin Content and Purity Determination

**Figure 11:**
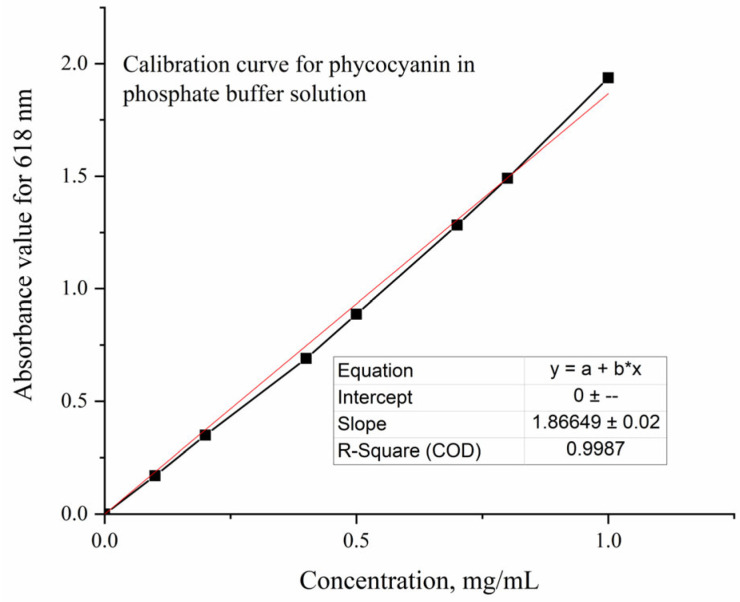
Calibration curve for phycocyanin in phosphate buffer solution.

To determine the amount of extracted interest compound, a calibration curve (Figure 11) was plotted using Blue Spirulina Powder dissolved in a phosphate buffer solution (pH = 7.4). For measurements, the Jasco V-550 UV-Vis Spectrophotometer (Jasco, Tokyo, Japan) was used and quartz cuvettes with 1 cm path length were utilized.

The resulting equation—Equation (1), was used for phycocyanin determination. The purity of the natural colorant was calculated using Equation (2) according to Liu et al. [56].

#### 3.3.2. Antioxidant Activity Determination

The antioxidant activity was determined according to the CUPRAC (CUPric Reducing Antioxidant Capacity) assay [87]. This analysis is based on the ability of the phenolic compounds to reduce Cu (II) ions. The used solutions, 10^–2^ M CuCl_2_, 1.0 M ammonium acetate (NH_4_Ac), and 7.5 × 10^−3^ M neocuproine (Nc), were prepared. For the sample analysis, 1 mL of each, together with x mL sample, and (1.1 − x) mL distilled water was added to a vial. After 30 min, the samples were analyzed using the Jasco V550 UV-VIS spectrophotometer, by measuring the absorbance value detected at 450 nm, against the control (all the reagents without the sample). The antioxidant activity was quantified as milligrams of Trolox equivalents (TE) per 1 g of dry matter (mg TE/g algae), using a standard curve corresponding to 0–0.25 mg Trolox/mL distilled water.

#### 3.3.3. FT-IR Analysis of the Extracts and of the Polymer-Alginate Hydrogels

Powder samples were obtained by lyophilization of the extracts using a Biobase BK-FD10S equipment and subjected FT-IR analysis on a Spectrum Two FTIR spectrometer (PerkinElmer, Waltham, MA, USA) equipped with a MIRacle^TM^ Single Reflection ATR (PIKE Technologies, Fitchburg, WI, USA), at 4 cm^−1^ resolution, totaling 32 scans, and 4000–550 cm^−1^ wavenumber range. The polymer-alginate hydrogels were dried and grinded for the FT-IR analysis performed in the same conditions.

#### 3.3.4. Scanning Electron Microscopy of the Blank Hydrogels and Loaded Hydrogels with Phycocyanin

The morphology of the polymer-alginate based hydrogels was performed after lyophilization of the specimens as described in Section 3.2.3. The micrograph images were obtained in cross-section and recorded at 10 kV through field emission gun scanning electron microscope (FEGSEM) Nova NanoSEM 630 (FEI) (Hillsboro, OR, USA) to analyze the interior of the specimens. The samples were covered with a thin Au layer (approx. 50 nm) to increase image resolution. The pore size distribution of the formulated specimens was statistically determined by measuring approximately 300 individual pores from SEM micrographs. The histograms, which revealed a unimodal distribution of the pores, were best fitted using the Gauss function (Figure S2).

#### 3.3.5. Compression Tests for the Polymer-Alginate-Based Hydrogels

To evaluate the mechanical properties of the hydrogels, uniaxial compressive tests were conducted on equilibrium-swelled samples. The tests were carried out using a Discovery TA instrument (Waters, Shanghai, China), equipped with parallel plane compression clamps (Ø 40 mm) as presented in Figure S3. The compression tests were performed using rate control-strain ramp mode at a speed of 1 mm/min. For each type of sample, five equilibrium-swollen disc-shaped specimens were tested, and the mean values were reported.

#### 3.3.6. Phycocyanin Release from Polymer-Alginate Based Hydrogels

A volume of 30 mL of simulated body fluid (SBF) with pH = 7.45 was placed in a 50 mL plastic vial and used as release medium. Prior to adding the hydrogel loaded with phycocyanin, the vial was positioned for 24 h in a HCM100-PRO Thermo Mix dry bath (Thermo, Waltham, MA, USA) set at 37 °C and 250 rpm. The same parameters were maintained for the controlled delivery of phycocyanin-loaded hydrogels over a 9 h period. Samples of 3 mL were collected every 10 min for the first 2 h, every 30 min for the following 3 h, and every 60 min up until 9 h. The removed release medium volume was supplemented with PBS, so a constant of 30 mL was retained. The samples were kept at 4–8 °C before being spectrophotometrically analyzed.

The amount of released phycocyanin was calculated according to Equation (4).

(5) $${CC}_{t=n}=\sum_{t=0}^{n-1}CC+Conc_{t=n}*30,$$

where: CC_t=n_ is the cumulative phycocyanin amount released at time t = n [mg], $\sum_{t=0}^{n}CC$ is the sum of the amount of phycocyanin released beforehand (taken out when sampling is performed) [mg/mL], and Conc_t=n_ is the phycocyanin concentration at time t = n [mg/mL] calculated according to Equation (2) (30 represents the volume used to perform the release studies).

## 4. Conclusions

In conclusion, phycocyanin active compound was extracted from *Spirulina platensis* by conventional, direct UAE, indirect UAE, and MAE extraction methods by varying different parameters like temperature, extraction time, stirring rate (conventional), and power intensity (in the case of UAE) to determine the optimum conditions in terms of highest phycocyanin concentration, antioxidant activity, and purity ratio. Although the maximum concentration of phycocyanin extract and highest value of antioxidant activity was obtained in the case of direct sonication of S. platensis, the peak purity of the extract was registered by MAE method.

Thus, phycocyanin loading of the hydrogels could be a suitable strategy for designing products for medical applications particularly in fields where structural integrity of the polymer-based matrix is required combined with moderate compression strength like tissue regeneration, drug delivery and wound dressings that can conform to body contours while ensuring cell proliferation, biocompatibility and structure integrity.

Considering the antioxidant properties of the phycocyanin extract, PAM/PVA-based alginate hydrogel formulations were proposed as the polymer matrix for phycocyanin release at two different pH levels (6.5 and 7.45). The release kinetics were evaluated using various models to identify the most suitable mechanism for drug release from the polymeric matrix. In our study, the release at pH 7.45 followed the Higuchi model most closely, while at pH 6.5, the same model also provided the best fit. The differences in release behavior between the two pH levels can largely be attributed to changes in phycocyanin solubility at different pH values.

## Figures and Tables

**Table 1:** Best outcomes for all the used extraction methods.

Extraction Method	Parameters	Phycocyanin Concentration, mg/mL	Antioxidant Activity, mg TE/g Alga	Rp
Indirect UAE	40 min, 40 °C, 72 W	16.95 ± 0.22	21.18 ± 0.16	0.32 ± 0.001
Direct UAE	Continuous, 30 min, 30 °C, 60 W	29.31 ± 0.33	20.15 ± 0.67	0.45 ± 0.003
	1 s ON 1 s OFF, 10 min, 30 °C, 30 W	22.45 ± 0.14	23.51 ± 0.38	0.37 ± 0.011
MAE	30 min, 40 °C, 900 rpm	13.82 ± 0.01	5.67 ± 0.11	0.5 ± 0.002
Conventional—CE-3	MAE reactor 50 min, 40 °C, 900 rpm	13.19 ± 0.47	5.25 ± 0.18	0.37 ± 0.002

**Table 2:** Antioxidant activity using CE methods and comparison with the unconventional equivalents.

Sample	Antioxidant Activity, mg TE/g Alga
CE-1	3.01 ± 0.10
UAE direct	20.15 ± 0.67
CE-2	4.78 ± 0.04
UAE indirect	21.17 ± 0.16
CE-3	5.26 ± 0.18
MAE	8.18 ± 0.07

**Table 3:** Parameters of the applied mathematical models at pH = 7.45, respectively at pH = 6.5.

Parameters of Mathematical Model	pH = 7.45	pH = 6.5
Zero Order (fi = K_0_·t)		
K_0_	0.0043	0.006
R^2^	0.9651	0.9181
$\mathrm{First}\;\mathrm{Order}\;(linearized\;logQ_{1}=logQ_{0}+\frac{k_{1\cdot t}}{2.303}$)		
Q_0_	0.4513	0.7863
k_1_	0.0085	0.0046
R^2^	0.9131	0.8802
Higuchi model ($f_{i}=K_{H}\cdot \sqrt{t})$		
K_H_	0.0404	0.0604
R^2^	0.9997	0.9872
Korsmeyer-Peppas (linearized equation log(fi) = log(k) + n·log(t))		
k	0.062	0.165
n	0.392	0.253
R^2^	0.9731	0.9811

## Data Availability

The original data presented in the study are included in the article/Supplementary Material; further inquiries can be directed to the corresponding author.

## Supplementary Materials

The following supporting information can be downloaded at: https://www.mdpi.com/article/10.3390/md22100434/s1, Figure S1: Images of the phycocyanin extract obtained by MAE (**a**), the polymer-alginate based hydrogel (**b**), the lyophilized hydrogel before loading (**c**), and the hydrogel after loading with phycocyanin (**d**); Figure S2: Mean size pores and pores distribution of the lyophilized hydrogel; Figure S3: Images during compression test of blank (**a**) and phycocyanin loaded hydrogels (**b**).

## References

[1] Tomaselli L., Vonshak A. (2002). Morphology, Ultrastructure and Taxonomy of *Arthrospira (Spirulina) maxima* and *Arthrospira (Spirulina) plantesis*. Spirulina Plantesis (Arthospira) Physiology, Cell-Biology and Biotechnology.

[2] Masojídek J., Torzillo G., Jørgensen S.E., Fath B.D. (2008). Mass Cultivation of Freshwater Microalgae. Encyclopedia of Ecology.

[3] Ho J.N., Watson R.R., Lee J., Watson R.R., Preedy V.R. (2013). Chapter 11—Dietary Supplements, Immune Modulation, and Diabetes Control. Bioactive Food as Dietary Interventions for Diabetes.

[4] Taufiqurrahmi N., Religia P., Mulyani G., Suryana D., Ichsan I., Tanjung F., Arifin Y. (2017). Phycocyanin extraction in Spirulina produced using agricultural waste. IOP Conf. Ser. Mater. Sci. Eng..

[5] Brito A.d.F., Silva A.S., de Oliveira C.V.C., de Souza A.A., Ferreira P.B., de Souza I.L.L., da Cunha Araujo L.C., da Silva Félix G., de Souza Sampaio R., Tavares R.L. (2020). *Spirulina platensis* prevents oxidative stress and inflammation promoted by strength training in rats: Dose-response relation study. Sci. Rep..

[6] Renugadevi K., Valli Nachiyar C., Sowmiya P., Sunkar S. (2018). Antioxidant activity of phycocyanin pigment extracted from marine filamentous cyanobacteria *Geitlerinema* sp. TRV57. Biocatal. Agric. Biotechnol..

[7] Grover P., Bhatnagar A., Kumari N., Narayan Bhatt A., Kumar Nishad D., Purkayastha J. (2021). C-Phycocyanin-a novel protein from *Spirulina platensis*—In vivo toxicity, antioxidant and immunomodulatory studies. Saudi J. Biol. Sci..

[8] Ravi M., Tentu S., Baskar G., Rohan Prasad S., Raghavan S., Jayaprakash P., Jeyakanthan J., Rayala S.K., Venkatraman G. (2015). Molecular mechanism of anti-cancer activity of phycocyanin in triple-negative breast cancer cells. BMC Cancer.

[9] Tantirapan P., Suwanwong Y. (2014). Anti-proliferative effects of C-phycocyanin on a human leukemic cell line and induction of apoptosis via the PI3K/AKT pathway. J. Chem. Pharm. Res..

[10] Khandual S., Sanchez E.O.L., Andrews H.E., de la Rosa J.D.P. (2021). Phycocyanin content and nutritional profile of *Arthrospira platensis* from Mexico: Efficient extraction process and stability evaluation of phycocyanin. BMC Chem..

[11] Pez Jaeschke D., Rocha Teixeira I., Damasceno Ferreira Marczak L., Domeneghini Mercali G. (2021). Phycocyanin from Spirulina: A review of extraction methods and stability. Food Res. Int..

[12] Su C.-H., Liu C.-S., Yang P.-C., Syu K.-S., Chiuh C.-C. (2014). Solid–liquid extraction of phycocyanin from *Spirulina platensis*: Kinetic modeling of influential factors. Sep. Purif. Technol..

[13] Jespersen L., Strømdahl L.D., Olsen K., Skibsted L.H. (2005). Heat and light stability of three natural blue colorants for use in confectionery and beverages. Eur. Food Res. Technol..

[14] Ferreira-Santos P., Nunes R., De Biasio F., Spigno G., Gorgoglione D., Teixeira J.A., Rocha C.M.R. (2020). Influence of thermal and electrical effects of ohmic heating on C-phycocyanin properties and biocompounds recovery from *Spirulina platensis*. LWT.

[15] Silveira S.T., Burkert J.F.M., Costa J.A.V., Burkert C.A.V., Kalil S.J. (2007). Optimization of phycocyanin extraction from *Spirulina platensis* using factorial design. Bioresour. Technol..

[16] Fernández-Rojas B., Hernández-Juárez J., Pedraza-Chaverri J. (2014). Nutraceutical properties of phycocyanin. J. Funct. Foods.

[17] Patil G., Raghavarao K.S.M.S. (2007). Aqueous two phase extraction for purification of C-phycocyanin. Biochem. Eng. J..

[18] Tavanandi H.A., Raghavarao K.S.M.S. (2020). Ultrasound-assisted enzymatic extraction of natural food colorant C-Phycocyanin from dry biomass of *Arthrospira platensis*. LWT.

[19] Acker J.P., McGann L.E. (2003). Protective effect of intracellular ice during freezing?. Cryobiology.

[20] Roquebert M.F., Bury E. (1993). Effect of freezing and thawing on cell membranes of Lentinus edodes, the shiitake mushroom. World J. Microbiol. Biotechnol..

[21] Tavanandi H.A., Mittal R., Chandrasekhar J., Raghavarao K.S.M.S. (2018). Simple and efficient method for extraction of C-Phycocyanin from dry biomass of Arthospira platensis. Algal Res..

[22] Chaiklahan R., Chirasuwan N., Loha V., Tia S., Bunnag B. (2011). Separation and purification of phycocyanin from *Spirulina* sp. using a membrane process. Bioresour. Technol..

[23] İlter I., Akyıl S., Demirel Z., Koç M., Conk-Dalay M., Kaymak-Ertekin F. (2018). Optimization of phycocyanin extraction from *Spirulina platensis* using different techniques. J. Food Compos. Anal..

[24] Pott R.W.M. (2019). The release of the blue biological pigment C-phycocyanin through calcium-aided cytolysis of live *Spirulina* sp.. Color. Technol..

[25] Jaeschke D.P., Mercali G.D., Marczak L.D.F., Müller G., Frey W., Gusbeth C. (2019). Extraction of valuable compounds from *Arthrospira platensis* using pulsed electric field treatment. Bioresour. Technol..

[26] Li Y., Zhang Z., Paciulli M., Abbaspourrad A. (2020). Extraction of phycocyanin—A natural blue colorant from dried spirulina biomass: Influence of processing parameters and extraction techniques. J. Food Sci..

[27] Käferböck A., Smetana S., de Vos R., Schwarz C., Toepfl S., Parniakov O. (2020). Sustainable extraction of valuable components from Spirulina assisted by pulsed electric fields technology. Algal Res..

[28] Larrosa A.P.Q., Camara Á.S., Moura J.M., Pinto L.A.A. (2018). *Spirulina* sp. biomass dried/disrupted by different methods and their application in biofilms production. Food Sci. Biotechnol..

[29] Moraes C.C., Sala L., Cerveira G.P., Kalil S.J. (2011). C-phycocyanin extraction from *Spirulina platensis* wet biomass. Braz. J. Chem. Eng..

[30] Pan-utai W., Iamtham S. (2019). Extraction, purification and antioxidant activity of phycobiliprotein from *Arthrospira platensis*. Process Biochem..

[31] Minchev I., Petkova N.T., Milkova-Tomova I. (2020). Ultrasound-Assisted Extraction of Chlorophylls and Phycocyanin from *Spirulina platensis*. Biointerface Res. Appl. Chem..

[32] Venugopal V.C., Thakur A., Chennabasappa L.K., Mishra G., Singh K., Rathee P., Ranjan A. (2020). Phycocyanin Extracted from Oscillatoria minima Shows Antimicrobial, Algicidal, and Antiradical Activities: In silico and In vitro Analysis. Anti-Inflamm. Anti-Allergy Agents Med. Chem..

[33] Thang N.H., Chien T.B., Cuong D.X. (2023). Polymer-Based Hydrogels Applied in Drug Delivery: An Overview. Gels.

[34] Wang Y., Malcolm D.W., Benoit D.S.W. (2017). Controlled and sustained delivery of siRNA/NPs from hydrogels expedites bone fracture healing. Biomaterials.

[35] Lei L., Bai Y., Qin X., Liu J., Huang W., Lv Q. (2022). Current Understanding of Hydrogel for Drug Release and Tissue Engineering. Gels.

[36] Ashley G.W., Henise J., Reid R., Santi D.V. (2013). Hydrogel drug delivery system with predictable and tunable drug release and degradation rates. Proc. Natl. Acad. Sci. USA.

[37] Caldwell A.S., Rao V.V., Golden A.C., Anseth K.S. (2020). Porous bio-click microgel scaffolds control hMSC interactions and promote their secretory properties. Biomaterials.

[38] Mastropietro D.J., Omidian H., Park K. (2012). Drug delivery applications for superporous hydrogels. Expert Opin. Drug Deliv..

[39] Foudazi R., Zowada R., Manas-Zloczower I., Feke D.L. (2023). Porous Hydrogels: Present Challenges and Future Opportunities. Langmuir.

[40] Fraser D., Nguyen T., Kotelsky A., Lee W., Buckley M., Benoit D.S.W. (2022). Hydrogel Swelling-Mediated Strain Induces Cell Alignment at Dentin Interfaces. ACS Biomater. Sci. Eng..

[41] Huang H., Dong Z., Ren X., Jia B., Li G., Zhou S., Zhao X., Wang W. (2023). High-strength hydrogels: Fabrication, reinforcement mechanisms, and applications. Nano Res..

[42] Olăreț E., Steinmüller-Nethl D., Iovu H., Stancu I.C. (2022). Nanodiamond Loaded Fish Gelatin Enzymatically Crosslinked Hydrogels. UPB Sci. Bull. Ser. B Chem. Mater. Sci..

[43] Simonca A.G., Albu Kaya M.G., Rău I., Marin M.M., Dinu-Pârvu C.-E., Lupuliasa A., Ghica M.V. (2023). The Influence of the Formulation Factors on the Design and Characterization of Some Collagen-Based Hydrogels with Metronidazole. UPB Sci. Bull. Ser. B Chem. Mater. Sci..

[44] Gherman S.P., Biliuță G., Bele A., Ipate A.M., Baron R.I., Ochiuz L., Șpac A.F., Zavastin D.E. (2023). Biomaterials Based on Chitosan and Polyvinyl Alcohol as a Drug Delivery System with Wound-Healing Effects. Gels.

[45] Ailincai D., Gavril G., Marin L. (2020). Polyvinyl alcohol boric acid—A promising tool for the development of sustained release drug delivery systems. Mater. Sci. Eng. C.

[46] Rivera-Hernández G., Antunes-Ricardo M., Martínez-Morales P., Sánchez M.L. (2021). Polyvinyl alcohol based-drug delivery systems for cancer treatment. Int. J. Pharm..

[47] Pavaloiu R.-D., Stoica-Guzun A., Stroescu M., Jinga S.I., Dobre T. (2014). Composite films of poly(vinyl alcohol)–chitosan–bacterial cellulose for drug controlled release. Int. J. Biol. Macromol..

[48] Kamoun E.A., Chen X., Mohy Eldin M.S., Kenawy E.-R.S. (2015). Crosslinked poly(vinyl alcohol) hydrogels for wound dressing applications: A review of remarkably blended polymers. Arab. J. Chem..

[49] Kamoun E.A., Loutfy S.A., Hussein Y., Kenawy E.-R.S. (2021). Recent advances in PVA-polysaccharide based hydrogels and electrospun nanofibers in biomedical applications: A review. Int. J. Biol. Macromol..

[50] Isopencu G., Deleanu I., Busuioc C., Oprea O., Surdu V.-A., Bacalum M., Stoica R., Stoica-Guzun A. (2023). Bacterial Cellulose—Carboxymethylcellulose Composite Loaded with Turmeric Extract for Antimicrobial Wound Dressing Applications. Int. J. Mol. Sci..

[51] Sahoo D.R., Biswal T. (2021). Alginate and its application to tissue engineering. SN Appl. Sci..

[52] Abourehab M.A.S., Rajendran R.R., Singh A., Pramanik S., Shrivastav P., Ansari M.J., Manne R., Amaral L.S., Deepak A. (2022). Alginate as a Promising Biopolymer in Drug Delivery and Wound Healing: A Review of the State-of-the-Art. Int. J. Mol. Sci..

[53] Bruschi M.L. (2015). Mathematical models of drug release. Strategies to Modify the Drug Release from Pharmaceutical Systems.

[54] Morgulchik N., Kamaly N. (2021). Meta-analysis of In Vitro Drug-Release Parameters Reveals Predictable and Robust Kinetics for Redox-Responsive Drug-Conjugated Therapeutic Nanogels. ACS Appl. Nano Mater..

[55] Adjali A., Clarot I., Chen Z., Marchioni E., Boudier A. (2022). Physicochemical degradation of phycocyanin and means to improve its stability: A short review. J. Pharm. Anal..

[56] Liu L.-N., Chen X.-L., Zhang X.-Y., Zhang Y.-Z., Zhou B.-C. (2005). One-step chromatography method for efficient separation and purification of R-phycoerythrin from *Polysiphonia urceolata*. J. Biotechnol..

[57] Gorgich M., Passos M.L.C., Mata T.M., Martins A.A., Saraiva M.L.M.F.S., Caetano N.S. (2020). Enhancing extraction and purification of phycocyanin from *Arthrospira* sp. with lower energy consumption. Energy Rep..

[58] Ferreira da Silva A., Brazinha C., Costa L., Caetano N.S. (2020). Techno-economic assessment of a Synechocystis based biorefinery through process optimization. Energy Rep..

[59] Sukor N., Jusoh R., Rahim S.A., Kamarudin N. (2018). Ultrasound assisted methods for enhanced extraction of phenolic acids from Quercus Infectoria galls. Mater. Today Proc..

[60] Aznar-Ramos M.J., Razola-Díaz M.d.C., Verardo V., Gómez-Caravaca A.M. (2022). Comparison between Ultrasonic Bath and Sonotrode Extraction of Phenolic Compounds from Mango Peel By-Products. Horticulturae.

[61] Vinatoru M., Mason T.J., Calinescu I. (2017). Ultrasonically assisted extraction (UAE) and microwave assisted extraction (MAE) of functional compounds from plant materials. TrAC Trends Anal. Chem..

[62] Parker F.S. (1971). Amides and Amines. Applications of Infrared Spectroscopy in Biochemistry, Biology, and Medicine.

[63] Kong J., Yu S. (2007). Fourier Transform Infrared Spectroscopic Analysis of Protein Secondary Structures. Acta Biochim. Biophys. Sin..

[64] Chen Q., Li S., Xiong H., Zhao Q. (2022). Effect of Different Extraction Methods on Physicochemical Characteristics and Antioxidant Activity of C-Phycocyanin from Dry Biomass of *Arthrospira platensis*. Foods.

[65] Prabakaran G., Sampathkumar P., Kavisri M., Moovendhan M. (2020). Extraction and characterization of phycocyanin from *Spirulina platensis* and evaluation of its anticancer, antidiabetic and antiinflammatory effect. Int. J. Biol. Macromol..

[66] Al-Malki A.L. (2020). In vitro cytotoxicity and pro-apoptotic activity of phycocyanin nanoparticles from Ulva lactuca (Chlorophyta) algae. Saudi J. Biol. Sci..

[67] Jipa I., Stoica A., Stroescu M., Dobre L.-M., Dobre T., Jinga S., Tardei C. (2012). Potassium sorbate release from poly(vinyl alcohol)-bacterial cellulose films. Chem. Pap..

[68] Xiong C., Wei F., Li W., Liu P., Wu Y., Dai M., Chen J. (2018). Mechanism of Polyacrylamide Hydrogel Instability on High-Temperature Conditions. ACS Omega.

[69] Gaabour L.H. (2017). Spectroscopic and thermal analysis of polyacrylamide/chitosan (PAM/CS) blend loaded by gold nanoparticles. Results Phys..

[70] Belattmania Z., Kaidi S., El Atouani S., Katif C., Bentiss F., Jama C., Reani A., Sabour B., Vasconcelos V. (2020). Isolation and FTIR-ATR and 1H NMR Characterization of Alginates from the Main Alginophyte Species of the Atlantic Coast of Morocco. Molecules.

[71] Kłosiński K.K., Wach R.A., Girek-Bąk M.K., Rokita B., Kołat D., Kałuzińska-Kołat Ż., Kłosińska B., Duda Ł., Pasieka Z.W. (2023). Biocompatibility and Mechanical Properties of Carboxymethyl Chitosan Hydrogels. Polymers.

[72] Vedadghavami A., Minooei F., Mohammadi M.H., Khetani S., Rezaei Kolahchi A., Mashayekhan S., Sanati-Nezhad A. (2017). Manufacturing of hydrogel biomaterials with controlled mechanical properties for tissue engineering applications. Acta Biomater..

[73] Fitzgerald M.M., Bootsma K., Berberich J.A., Sparks J.L. (2015). Tunable Stress Relaxation Behavior of an Alginate-Polyacrylamide Hydrogel: Comparison with Muscle Tissue. Biomacromolecules.

[74] Darnell M.C., Sun J.-Y., Mehta M., Johnson C., Arany P.R., Suo Z., Mooney D.J. (2013). Performance and biocompatibility of extremely tough alginate/polyacrylamide hydrogels. Biomaterials.

[75] Sun L., Wang S., Qiao Z. (2006). Chemical stabilization of the phycocyanin from cyanobacterium *Spirulina platensis*. J. Biotechnol..

[76] Li Y., Li Q., Gillilan R.E., Abbaspourrad A. (2023). Reversible disassembly-reassembly of C-phycocyanin in pressurization-depressurization cycles of high hydrostatic pressure. Int. J. Biol. Macromol..

[77] Jing Z., Xu A., Liang Y.-Q., Zhang Z., Yu C., Hong P., Li Y. (2019). Biodegradable Poly(acrylic acid-co-acrylamide)/Poly(vinyl alcohol) Double Network Hydrogels with Tunable Mechanics and High Self-healing Performance. Polymers.

[78] Pereira L., Cotas J., Blumenberg M. (2020). Alginates: Recent Uses of This Natural Polymer.

[79] Xing M.-Y., Yu C.-L., Wu Y.-F., Wang L., Guan G.-P. (2020). Preparation and characterization of a polyvinyl alcohol/polyacrylamide hydrogel vascular graft reinforced with a braided fiber stent. Text. Res. J..

[80] Mircioiu C., Voicu V., Anuta V., Tudose A., Celia C., Paolino D., Fresta M., Sandulovici R., Mircioiu I. (2019). Mathematical Modeling of Release Kinetics from Supramolecular Drug Delivery Systems. Pharmaceutics.

[81] Merchant H.A., Shoaib H.M., Tazeen J., Yousuf R.I. (2017). Once-daily tablet formulation and in vitro release evaluation of cefpodoxime using hydroxypropyl methylcellulose: A technical note. AAPS PharmSciTech.

[82] Zhu W., Long J., Shi M. (2023). Release Kinetics Model Fitting of Drugs with Different Structures from Viscose Fabric. Materials.

[83] Debreczeny M., Gombos Z., Csizmadia V., Várkonyl Z., Szalontai B. (1989). Chromophore conformational analysis in phycocyanin and in related chromopeptides by surface enhanced Raman spectroscopy. Biochem. Biophys. Res. Commun..

[84] Falkeborg M.F., Roda-Serrat M.C., Burnæs K.L., Nielsen A.L.D. (2018). Stabilising phycocyanin by anionic micelles. Food Chem..

[85] Li Y., Zhang Z., Abbaspourrad A. (2022). Improving solubility and functional properties of phycocyanin under acidic conditions by glutaminase deamidation and succinylation. Food Hydrocoll..

[86] Saraydın D., Karadaǧ E., Işıkver Y., Şahiner N., Güven O. (2004). The Influence of Preparation Methods on the Swelling and Network Properties of Acrylamide Hydrogels with Crosslinkers. J. Macromol. Sci. Part A.

[87] Özyürek M., Güçlü K., Tütem E., Başkan K.S., Erçağ E., Esin Çelik S., Baki S., Yıldız L., Karaman Ş., Apak R. (2011). A comprehensive review of CUPRAC methodology. Anal. Methods.

