# Supplementary material for: Phycocyanin-Loaded Alginate-Based Hydrogel Synthesis and Characterization
Source: Mar Drugs. 2024 Sep 25;22(10):434. doi: 10.3390/md22100434 (PMC11509733; doi:10.3390/md22100434)
Supplement: Supplementary file 1 [file marinedrugs-22-00434-s001.zip › marinedrugs-3227225-supplementary.pdf]

marine drugs

Supplementary Information

# Phycocyanin-Loaded Alginate-Based Hydrogel Synthesis and Characterization

**Diana-Ioana Buliga [1], Alexandra Mocanu [1,2], Edina Rusen [1], Aurel Diacon [3], Gabriela Toader [3], Oana Brincoveanu [2,4], Ioan Călinescu [1] and Aurelian Cristian Boscornea [1,*]**

1 Faculty of Chemical Engineering and Biotechnologies, National University of Science and Technology POLITEHNICA Bucharest, 1-7 Gheorghe Polizu St., 1st District, 011061 Bucharest, Romania; diana_ioana.buliga@upb.ro (D.-I.B.); alexandra.mocanu@imt.ro (A.M.); edina.rusen@upb.ro (E.R.); ioan.calinescu@upb.ro (I.C.)
2 National Institute for Research and Development in Microtechnologies-IMT Bucharest, 126A Erou Iancu Nicolae, 077190 Voluntari, Romania; oana.brincoveanu@imt.ro
3 Military Technical Academy "Ferdinand I", 39-49 G. Cosbuc Blvd., 050141 Bucharest, Romania; aurel.diacon@mta.ro (A.D.); gabriela.toader@mta.ro (G.T.)
4 Research Institute, University of Bucharest, ICUB Bucharest, 90 Panduri Rd., 5th District, 050663 Bucharest, Romania
* Correspondence: cristian.boscornea@upb.ro

**Citation:** Buliga, D.-I.; Mocanu, A.; Rusen, E.; Diacon, A.; Toader, G.; Brincoveanu, O.; Călinescu, I.; Boscornea, A.C. Phycocyanin-Loaded Alginate-Based Hydrogel Synthesis and Characterization. *Mar. Drugs* **2024**, *22*, 434. https://doi.org/10.3390/md22100434

Academic Editors: Laurent Picot and Raimundo Gonçalves de Oliveira Junior

Received: 10 September 2024
Revised: 23 September 2024
Accepted: 24 September 2024
Published: 25 September 2024

**Copyright:** © 2024 by the authors. Licensee MDPI, Basel, Switzerland. This article is an open access article distributed under the terms and conditions of the Creative Commons Attribution (CC BY) license (https://creativecommons.org/licenses/by/4.0/).

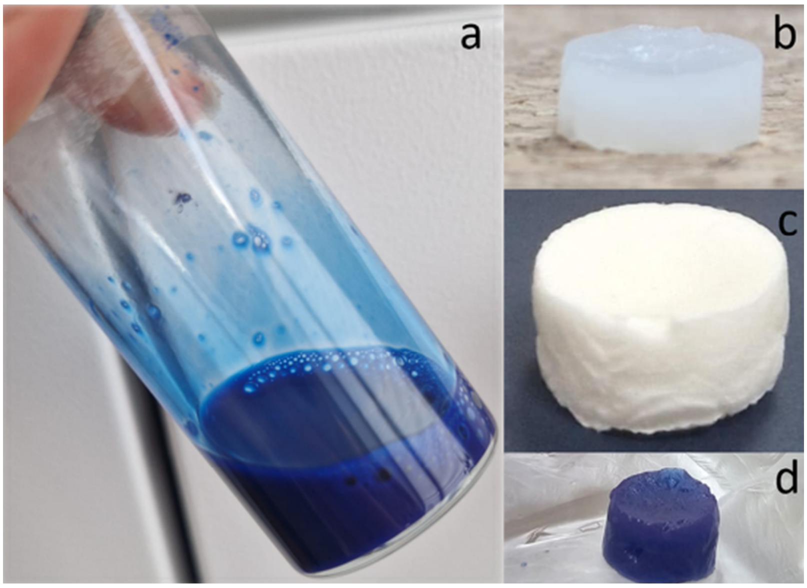


**Figure S1.** Images of the phycocyanin extract obtained by MAE (**a**), the polymer-alginate based hydrogel (**b**), the lyophilized hydrogel before loading (**c**), and the hydrogel after loading with phycocyanin (**d**).

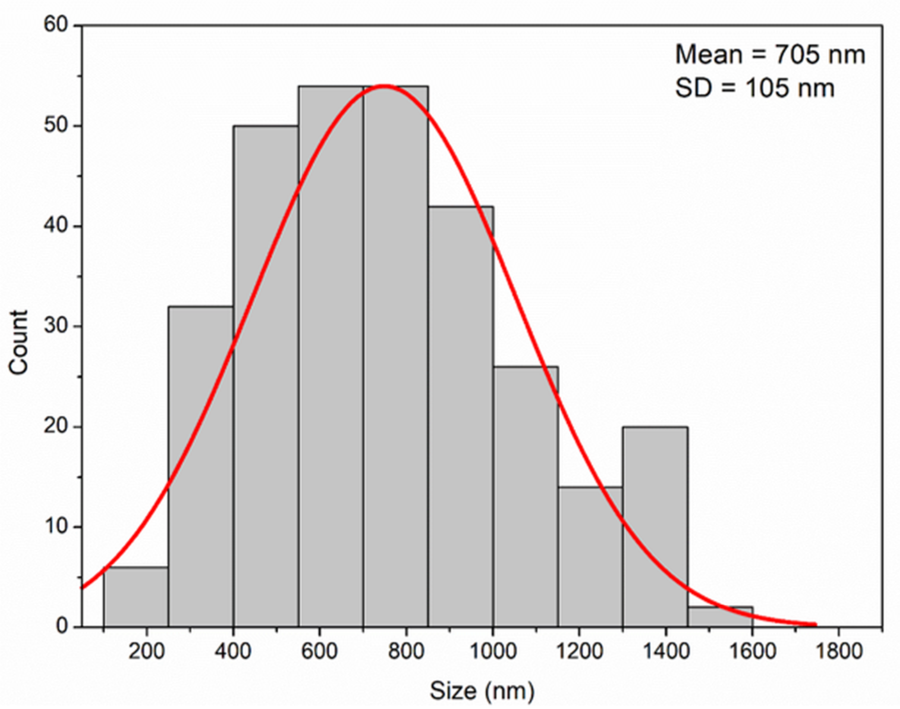


**Figure S2.** Mean size pores and pores distribution of the lyophilized hydrogel

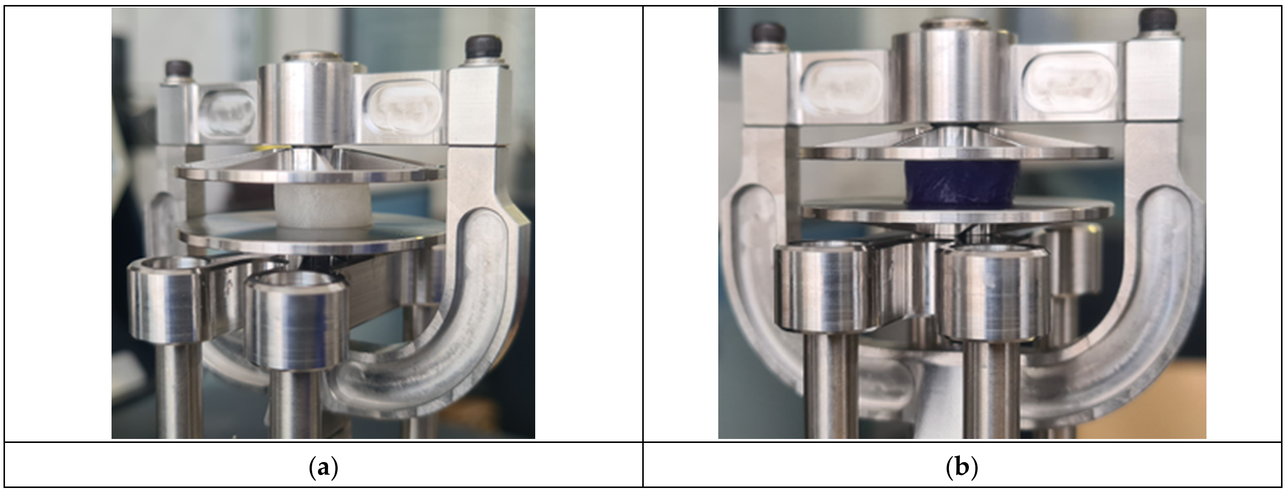

| (**a**) | (**b**) |
|---|---|

**Figure S3.** Images during compression test of blank (**a**) and phycocyanin loaded hydrogels (**b**)
